# High-salt microenvironment worsens the progression of atopic dermatitis via activating the SGK–1–mTOR pathway in keratinocytes

**DOI:** 10.1016/j.gendis.2026.102168

**Published:** 2026-03-27

**Authors:** Yingqiang Luo, Zhiqiang Song, Pengju Jiang, Lan Ge, Min Zhang, Zihao Zhou, Yaguang Wu, Jun Hu

**Affiliations:** a Department of Dermatology, Southwest Hospital, Third Military Medical University (Army Medical University), Chongqing 400038, China; b Department of Neurology, Southwest Hospital, Third Military Medical University (Army Medical University), Chongqing 400038, China

**Keywords:** Atopic dermatitis, Keratinocytes, SGK-1, Skin barrier, Sodium chloride

## Abstract

Atopic dermatitis (AD) is a prevalent chronic skin disease worldwide. Emerging evidence suggests that an elevated salt microenvironment is a unique characteristic of skin lesions in AD patients. Emerging data indicate that lesional skin in AD contains abnormally high salt, yet how this microenvironment affects keratinocytes is unknown. We interrogated the impact of elevated NaCl on barrier function and inflammation using HaCaT cells *in vitro*, and 2,4-dinitrofluorobenzene (DNFB) and ovalbumin (OVA) treatment induced an AD model of mice *in vivo*. The interaction between SGK-1, mTOR, STAT3, and NFκB was identified with their roles in AD, analyzed through loss-of-function assays and pharmacological inhibitions. Our results showed that the high-salt microenvironment impaired keratinocyte differentiation and promoted inflammatory cytokine production, thereby exacerbating the disease. Mechanistically, NaCl activated STAT3 via the SGK-1–mTOR signaling pathway in keratinocytes, leading to a decrease in the expression of epidermal barrier proteins. In addition, inflammatory cytokines were increased by NaCl-induced SGK-1–mTOR–NFκB activation. We identified the SGK-1 inhibitor GSK 650394, which could rescue the NaCl-induced blockade of keratinocyte differentiation and inflammatory cytokine production. Moreover, in combination with the JAK inhibitor delgocitinib, GSK 650394 exhibits a synergistic effect in ameliorating AD-like symptoms *in vivo*. Taken together, our findings demonstrate that the high-salt microenvironment promotes the development of AD by activating the SGK-1–mTOR pathway in keratinocytes, suggesting that an SGK-1 inhibitor could potentially serve as a drug to alleviate AD-like symptoms.

## Background

Atopic dermatitis (AD), also known as atopic eczema, is a common and long-lasting skin condition that primarily manifests as severe itching and inflammation.^1^^,^^2^ Epidemiologic studies have shown that AD affects a significant proportion of the world's population, with the prevalence rates ranging from 15% to 20% in children and 10% in adults.^3^ The impact of AD on patients' quality of life cannot be overstated, as it often leads to allergic rhinitis and asthma, a phenomenon referred to as the atopic march.^4^ In addition, AD places a heavy financial burden on healthcare systems worldwide due to its high medical costs, making it the most economically burdensome skin disease. In recent decades, the incidence and prevalence of AD have shown a worrying upward trend. This increase is largely due to environmental factors, including exposure to air pollutants and the widespread use of household hygiene products.^5^^,^^6^ These environmental stimuli contribute to the complex pathogenesis of AD, which is characterized by an exaggerated immune response mainly mediated by *T* helper type 2 (Th2) cells. Th2 cells are known to produce various cytokines that promote inflammation, such as interleukin (IL)-4 and IL-13, leading to the characteristic symptoms of AD. Biologics such as anti-IL-4 or anti-IL-13 antibodies have been approved for use in patients with moderate to severe AD, but limitations such as the development of resistance and high costs still exist.^7^ Therefore, it is necessary to develop a new AD therapy based on a deeper understanding of the pathophysiology of AD to minimize the side effects and provide complete disease remission.

In recent years, a growing body of evidence has supported the idea that skin barrier dysfunction plays a critical role in the initiation and progression of AD.^8^ The skin's epidermal barrier function relies primarily on the outermost layer of the epidermis, which is composed of terminally differentiated keratinocytes.^9^ To form a robust and intact epidermal barrier, keratinocytes undergo a highly regulated process of differentiation. They gradually progress from the stratum basale (SB) layer to the stratum spinous (SS) layer, stratum granular (SG) layer, and finally to the stratum corneum (SC) layer of the epidermis.^10^ In AD patients, skin lesions are characterized by a severe impairment of the terminal differentiation of keratinocytes in the SC layer. This is accompanied by an expansion of cells in the SB layer and a reduction of cells in the SS and SG layers.^11^ The failure of terminal differentiation in keratinocytes leads to a decrease in skin barrier proteins, such as filaggrin (FLG) and loricrin (LOR), in typical skin lesions of AD patients.^12^ In addition, keratinocytes have the ability to produce specific inflammatory chemokines and cytokines as well. Among these, thymic stromal lymphopoietin (TSLP), produced particularly by keratinocytes, plays a critical role in the pathogenesis of AD.^13^ TSLP is not detectable in normal or diseased skin, but its expression is significantly increased in the acute and chronic lesions of AD patients. TSLP acts on dendritic cells to create a microenvironment that induces the differentiation of inflammatory Th2 cells.^14^ In addition, TSLP has been found to stimulate mast cells, leading to the production of high levels of Th2 cytokines through the activation of mouse double minute 2 (MDM2) and signal transducer and activator of transcription 6 (STAT6).^15^ Keratinocytes are also an important source of other cytokines, such as IL-25 and IL-33, in skin lesions. These cytokines have a profound effect on T cells and contribute to the pathogenesis of various skin diseases.^13^ Taken together, these studies suggest that keratinocytes are important regulatory and executive cells in AD pathogenesis, but the mechanism underlying AD-induced differentiation blockade and inflammatory cytokine production in keratinocytes is poorly understood.

Recent studies have revealed a fascinating discovery about the skin lesions of AD patients. These lesions were found to have a specific high-salt (sodium chloride) microenvironment compared with the normal skin of AD patients and skin lesions of other skin diseases, such as psoriasis. This suggests that the localized high-salt microenvironment is an exclusive feature of AD lesions.^16^ This localized high-salt microenvironment in AD lesions may indeed play a significant role in the initiation and development of AD. Further research has shown that the high levels of sodium chloride (NaCl) in the AD microenvironment can trigger the differentiation of Th0 cells into Th2 cells by activating the transcription factor nuclear factor of activated T cells 5 (NFAT5), and this activation of Th2 cells exacerbates disease progression.^16^ However, the impact of this local high-NaCl microenvironment on keratinocytes, the predominant cell type in the epidermis, remains unclear. In this study, we aimed to shed light on this aspect and discovered that the high NaCl microenvironment in AD indeed inhibited keratinocyte differentiation. In addition, NaCl induced the production of inflammatory cytokines, which further promoted disease progression.

We investigate the underlying mechanisms and find that NaCl activates the signal transducer and activator of transcription 3 (STAT3) signaling pathway through the Serum and glucocorticoid regulated kinase 1 (SGK-1)–mammalian target of rapamycin (mTOR) pathway, leading to the suppression of keratinocyte differentiation and decreases in production of epidermal barrier proteins. Furthermore, this activation of SGK-1–mTOR also leads to increased production of inflammatory cytokines derived from keratinocytes due to activation of NF-κB. Excitingly, we also identified an SGK-1 inhibitor, GSK 650394, that could counteract the inhibitory effect of NaCl on keratinocyte differentiation and cytokine secretion both *in vitro* and *in vivo*. Furthermore, GSK 650394 exhibited a synergistic effect when combined with the clinically available pan-JAK inhibitor delgocitinib to alleviate AD-like dermatitis *in vivo*.

In conclusion, our findings provide critical insights into the role of the high-salt microenvironment in promoting the development of AD through activation of the SGK-1–mTOR pathway in keratinocytes. This opens up opportunities for the development of novel therapeutic interventions targeting this pathway and highlights the potential of combining SGK-1 and JAK inhibitors for the treatment of AD.

## Materials and methods

### Ethics statement

BALB/c mice were provided by the Department of Experimental Animals of the Army Medical University, and all were female, weighing about 25 g. The mice were housed according to a 12 h circadian rhythm with a relative humidity of 50% and an ambient temperature of 25 °C. Animal experiments were approved by the Ethics Committee of Southwest Hospital, and all operations were performed in accordance with relevant ethical regulations.

### Cell lines and cell culture

Human keratinocyte cell HaCaT was purchased from the Cell Bank of the Chinese Academy of Sciences (Shanghai, China). The cells were cultured using DMEM culture medium (HyClone, USA) supplemented with 10% fetal bovine serum (ABW, Uruguay) and 1% penicillin/streptomycin (Beyotime, China). All cells were cultured at 37 °C in a 5% CO_2_ incubator.

### Cell viability and apoptosis assay

NaCl was added to the DMEM culture medium at a concentration of 40 mM to achieve an osmolality of 380 mOsm/kg H_2_O. Similarly, mannitol, sodium gluconate (Na-Gluconate), and choline chloride (Choline-Cl) were prepared to reach the same osmolality of 380 mOsm/kg H_2_O, ensuring isotonic conditions. After 24 h of treatment with the prepared isotonic media, 10 μL of cell counting kit-8 (ShenEr, China) was added, and the optical density was measured at 450 nm to evaluate cell viability. Additionally, cells were collected after treatment and stained using the Annexin V-FITC/PI apoptosis detection kit (Beyotime, China). Apoptosis levels were then immediately detected using flow cytometry.

### The AD mouse model

Six-week-old female BALB/c mice weighing 20–25 g were procured from the Experimental Animal Centre of the Army Medical University (Third Military Medical University, Chongqing, 400,038, China). All the animals were housed in polypropylene cages and were given unlimited access to sterilized fodder and water. All experimental procedures were approved by the Laboratory Animal Welfare and Ethics Committee of Army Medical University (AMUWEC20223374). Animals were acclimatized to feeding conditions for 1 week before experimentation. At specific time points, the mice were sacrificed by cervical dislocation. After acclimation to the laboratory conditions, the back skin hair of all the mice was shaved with an electric razor, and the bare skin was treated with hair removal cream. The application of hair removal cream was repeated every three days to keep the skin clean. One hundred microliters of DNFB or solvent was uniformly smeared onto the shaved back of the mice three times a week. One hundred microliters of ovalbumin or saline was given twice a week, once for application to the shaved back skin, and once for intraperitoneal injection. Animals were sacrificed 24 h after the last exposure to reagents for the procurement of blood samples and skin tissue. All reagents were softly and evenly applied to the mice. Cages were sanitized and replenished with chow and water weekly.

### Enzyme-linked immunosorbent assay (ELISA)

Skin tissue samples were weighed and homogenized on ice. The homogenates were centrifuged at 12,000 rpm at 4 °C for 15 min, and the supernatants were taken for cytokine assay. The concentrations of IL-4, IL-13, IL-25, IL-33, TSLP, and IgE (R&D Systems, USA) in the tissue homogenates were quantified using ELISA kits according to the manufacturer's instructions. HaCaT cells were inoculated in six-well culture plates. After stimulation with reagents, cell homogenates were taken, and a cytokine assay was performed according to the manufacturer's instructions. The concentrations of IL-25, IL-33, and TSLP in HaCaT cell homogenates were measured by ELISA kits according to the reagent instructions.

### Western blotting

Cells were collected and lysed with RIPA buffer (Beyotime, China) containing protease inhibitors on ice for 30 min. Total proteins were quantified by the BCA Kit (Beyotime, China) according to the reagent instructions. Proteins were separated by gel electrophoresis and transferred to PVDF membranes. PVDF membranes were blocked in 5% bovine serum albumin for 2 h and incubated with primary antibody (1:1000) at 4 °C overnight. The membranes were then incubated with the secondary antibody for 1 h at room temperature and developed.

The primary antibodies used were against the following antigens: FLG (SCBT, USA), LOR (Thermofisher, USA), GAPDH (ShenEr, China), β-actin (Proteintech, China), NFAT5 (Affinity, USA), p38 MAPK (Zenbio, China), 4EBP1 (Proteintech, Chin), phospho-4EBP1 (Zenbio, China), 70S6K (CST, USA), phospho-70S6K (CST, USA), RPS6 (BIOSS, China), Phospho-RPS6 (BIOSS, China) (phospho-p38 MAPK (Zenbio, China), phospho-SGK-1 (Affinity, USA), phospho-SGK-1 (BIOSS, China), NFκB p65 (BIOSS, China), NFκB p50 (BIOSS, China), STAT3 (BIOSS, China), and phospho-STAT3 (BIOSS, China).

### Immunofluorescence

Cells were cultured in 12-well slides. Cells were fixed with 100% acetone at 4 °C for 10 min and then permeabilized with 0.1% Triton X-100 at room temperature for 10 min. Fixed cells were treated with 1% bovine serum albumin. Incubated with phosphate-buffered saline (PBS) for 1 h and with FLG and LOR antibodies for 1 h. The cells were then incubated with FITC-labeled antibody for 1 h. Images were captured using fluorescence microscopy after DAPI staining.

### Hematoxylin–eosin staining

Mouse skin tissues were fixed in 4% paraformaldehyde for 24 h, rinsed, dehydrated, and paraffin-embedded. The tissues were sectioned. Sections of 4–5 μm thickness were stained with hematoxylin–eosin staining kit (Solarbio, China) and then observed under a microscope.

### Skin lesion assessment

The lesion scores were recorded for each group of mice except the blank group, and the severity of lesions was scored with reference to the five main components: erythema, desquamation, dryness/scarring, edema, and exudation. Each component has four levels: 0 (none), 1 (mild), 2 (moderate), or 3 (severe). The total score for dermatitis was calculated based on the total score of these individuals. The scores for each project were evaluated by two independent observers. Skin damage assessment was conducted every four days and every 8 day at the end of the experiment. The water content of the stratum corneum, trans-epidermal water loss (TEWL), and sebum content (measured in μg/cm^2^) were measured using a non-invasive skin tester. The same area was selected for each measurement, and a total of three measurements were taken and averaged.

### Statistical analysis

GraphPad Prism software was used for statistical analysis, and the results of the statistical analysis were expressed as mean ± standard deviation. Student's *t*-test was used for comparison between two groups. One-way ANOVA and LSD *t*-test were used for analysis of differences between groups. *P*-value < 0.05 indicates statistical significance.

## Results

### The high-salt microenvironment promotes AD-like dermatitis development in murine models

**Figure 1:**
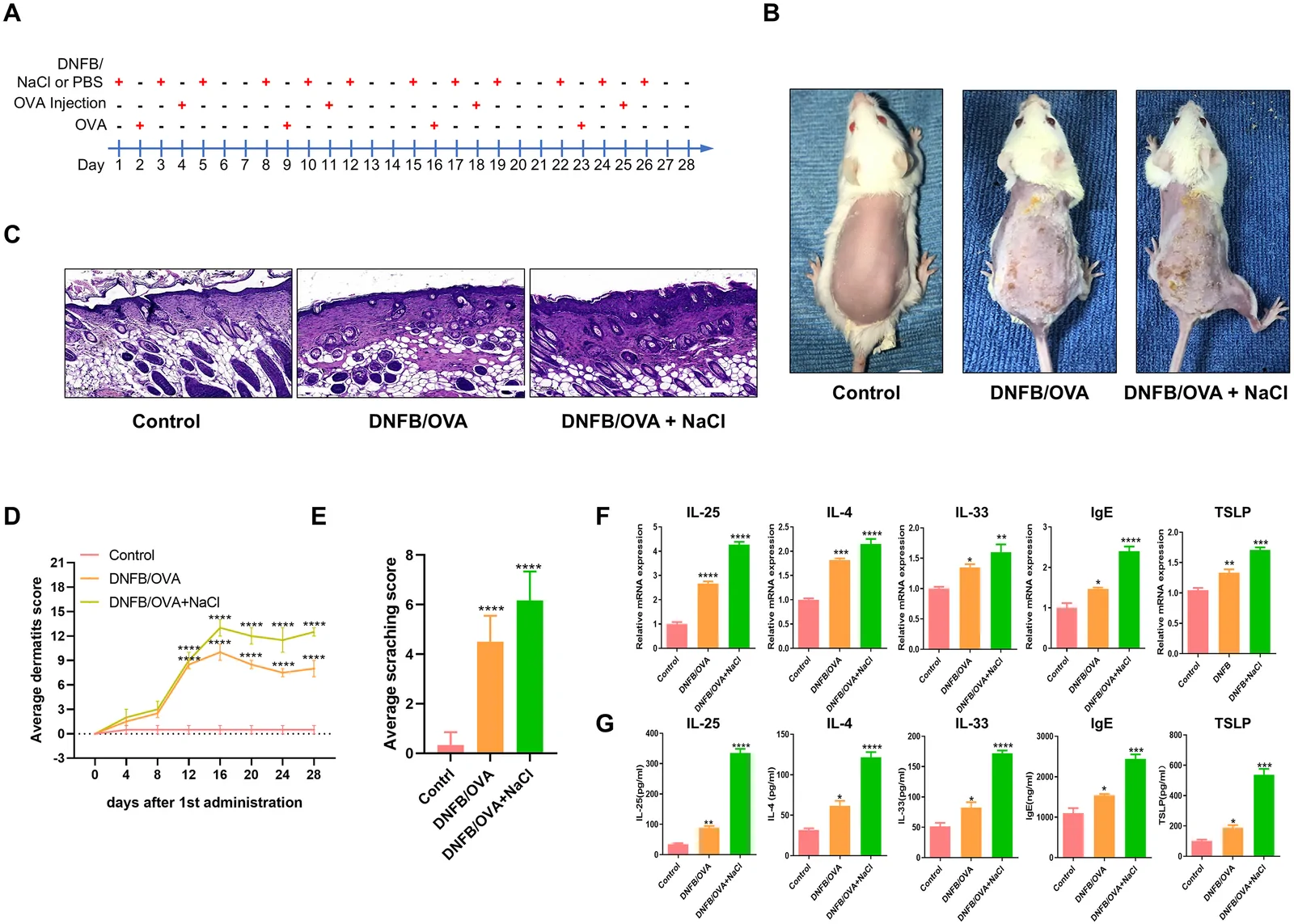
The high-salt microenvironment promotes disease development in mice with DNCB/OVA-induced atopic dermatitis **(A)** Schematic of the animal experiment procedure **(B)** Representative photos of dorsal skin in DNCB/OVA-induced atopic dermatitis in PBS- or NaCl-treated mice **(C)** Representative mouse model skin tissues are shown by hematoxylin–eosin staining **(D)** Time course of total skin lesion scores of PBS- or NaCl-treated mice (*n* = 5) **(E)** Scratching behavior examination **(F)** Quantitative reverse transcription PCR assay of the related target mRNA levels in the dorsal skin lesion. ∗*p* < 0.05, ∗∗*p* < 0.01, and ∗∗∗*p* < 0.001 **(G)** Related target molecules secretion (ELISA) of the mouse model blood (*n* = 5). Statistical analysis was performed using Student's *t*-test. Data are expressed as mean ± standard deviation. ∗∗*p* < 0.01 and ∗∗∗*p* < 0.001.

To investigate the potential impact of a high-salt microenvironment on the development of AD, we conducted a study using AD mouse models. To establish these models, we subjected BALB/c mice to continuous treatment with DNFB and OVA for four consecutive weeks according to previously reported methods.^17^ Simultaneously, a smear of PBS or NaCl solution was applied to the backs of the mice representing the control and treatment groups, respectively (Fig. 1A). After the four-week treatment period, we photographed the mice and then sacrificed them to assess the effects of the NaCl solution on the development of AD. As shown in Figure 1B and C, NaCl-treated mice exhibited more pronounced and histologically evident skin lesions compared with PBS-treated mice. Furthermore, the dermatitis scores and scratching scores were significantly higher in NaCl-treated mice compared with their control counterparts (Fig. 1D and E). In addition, we observed a significant up-regulation of AD-related inflammatory cytokines (IL-4, IL-25, IL-33, IgE, and TSLP) at both the mRNA and protein levels in the NaCl-treated mice, as shown in Figure 1E and F. Taken together, these findings provide strong evidence that a high-salt microenvironment can indeed promote the development of AD *in vivo*.

### NaCl disrupts epidermal barrier function in AD and suppresses keratinocyte differentiation

**Figure 2:**
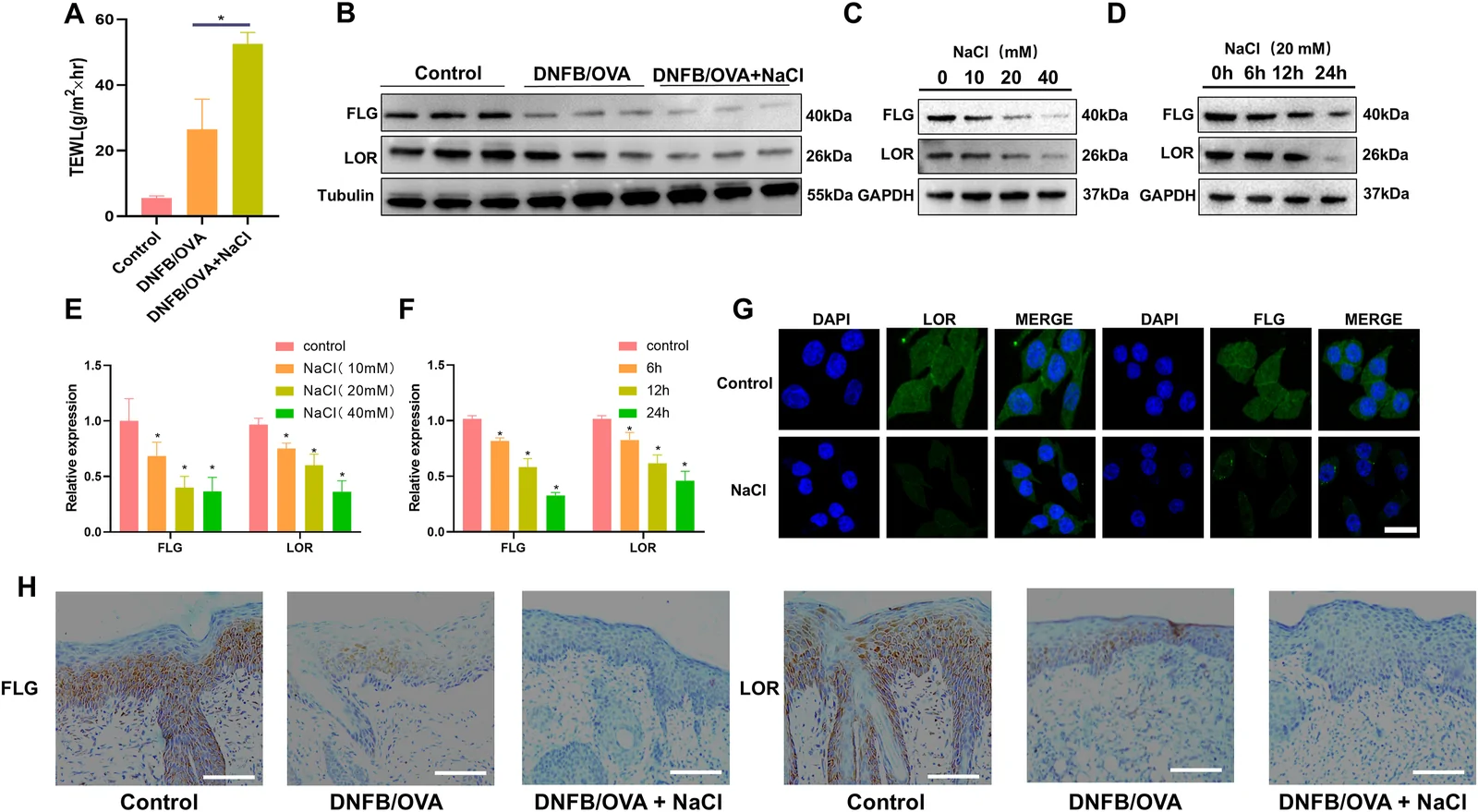
NaCl disrupts skin barrier function in atopic dermatitis and suppresses keratinocyte differentiation **(A)** Trans-epidermal water loss (TEWL) values of mice dorsal skin (*n* = 5) **(B)** Immunoblotting analysis of filaggrin (FLG) and loricrin (LOR) expression levels in skin lesions **(C)** Western blotting analysis of FLG and LOR expression levels at the indicated concentrations of NaCl in keratinocytes for 24 h **(D)** Western blotting analysis of FLG and LOR expression levels at different time points in keratinocytes treated with 20 mM NaCl **(E)** Quantitative reverse transcription PCR assay analysis of FLG and LOR mRNA expression levels in keratinocytes treated with different concentrations of NaCl (0 mM, 10 mM, 20 mM, 40 mM) **(F)** Quantitative reverse transcription PCR assay analysis of FLG and LOR mRNA expression levels at different time points in keratinocytes treated with 20 mM NaCl **(G)** Representative images of immuno-fluorescent staining of lysosome (stained by Lysotracker, red) and LOR or FLG (green) in keratinocytes treated with PBS and 20 mM NaCl. Scale bar: 10 μm **(H)** Representative immunohistochemistry images of skin lesions for LOR and FLG in PBS- or NaCl-treated mice. Scale bar: 50 μm. Data are shown as mean ± standard deviation from at least three independent experiments. Statistical analysis was performed using Student's *t*-test. Data are expressed as mean ± standard deviation. ∗∗*p* < 0.01 and ∗∗∗*p* < 0.001.

Skin barrier dysfunction is increasingly recognized as a pivotal factor in the pathogenesis of AD, with keratinocytes playing a critical role in maintaining skin barrier homeostasis.^8^^,^^13^ To further understand the mechanisms behind the progression of AD induced by a high-salt microenvironment, we conducted experiments to evaluate the skin barrier function. The results showed an increase in TEWL levels in the NaCl-treated mice, indicating a disruption of skin barrier function (Fig. 2A). Furthermore, Western blotting analysis revealed a significant decrease in the expression of the epidermal barrier proteins FLG and LOR in the NaCl-treated AD mice (Fig. 2B). To investigate the effects of high-salt microenvironment on keratinocytes *in vitro*, we treated the human keratinocyte cell line HaCaT with different concentrations of NaCl. The results obtained by quantitative PCR and Western blotting assays showed that both mRNA and protein levels of FLG and LOR decreased in a dose- and time-dependent manner (Fig. 2C–F). Furthermore, the inhibitory effects of NaCl on FLG and LOR expression were confirmed by immunofluorescence assays *in vitro* (Fig. 2G) and immunohistochemistry assay results *in vivo* (Fig. 2H). Taken together, these results indicate that NaCl suppresses keratinocyte differentiation and disrupts skin barrier function in AD.

### Cell viability and apoptosis in keratinocytes treated with NaCl

HaCaT cells were exposed to solutions containing or devoid of mannitol, NaCl, Na-gluconate, and choline chloride for 24 h, after which cell viability was evaluated. The findings indicated that, in comparison to the control group, treatment with the four hyperosmotic solutions at 380 mOsm/kg H_2_O did not significantly impact cell viability (Fig. S1A). Furthermore, apoptosis levels were analyzed by flow cytometry, and the high percentage of viable cells across all groups suggested that the cells maintained normal viability (Fig. S1B). These findings indicate that 380 mOsm/kg H_2_O and the associated ionic conditions are biocompatible with HaCaT cells. Additionally, we examined the effects of different isotonic solutions on keratinocyte barrier proteins (Fig. S2). The results revealed that, under 380 mOsm/kg H_2_O conditions, the levels of osmotic pressure-related proteins, including NFAT5 and phosphorylated p38, were significantly elevated. In contrast, the expression of barrier proteins was reduced in the NaCl group, and similar effects were observed in the sodium and chloride ion exchange groups. These findings suggest that the impact of NaCl on skin barrier function is not due to osmotic effects but rather to the specific microenvironmental changes induced by NaCl.

### NaCl activates the SGK-1–STAT3 pathway in keratinocytes

**Figure 3:**
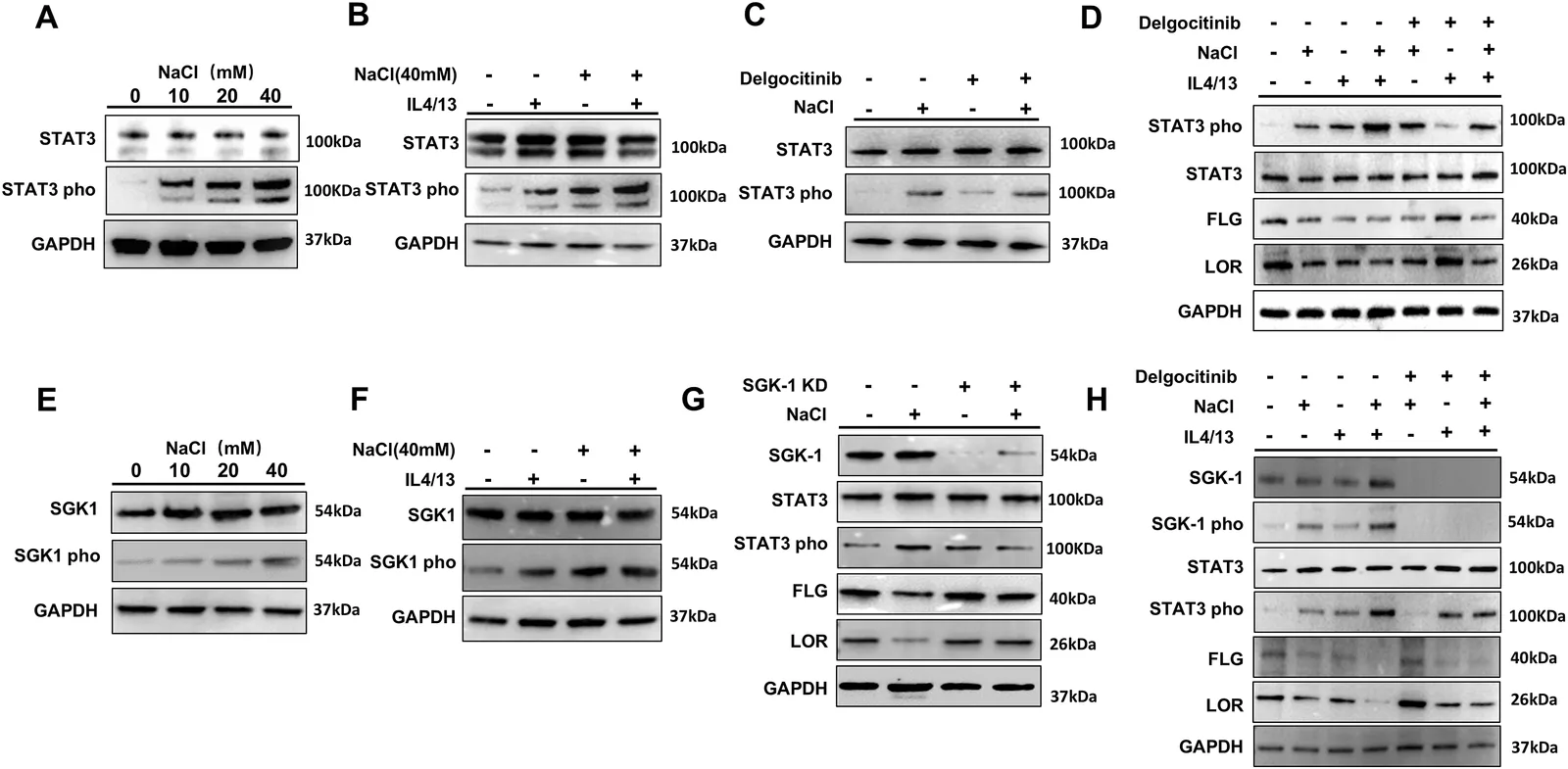
NaCl activates the SGK-1–STAT3 pathway in keratinocytes **(A)** Western blotting analysis of STAT3 and phosphorylation of STAT3 protein expression levels in keratinocytes were treated with different concentrations of NaCl (0 mM, 10 mM, 20 mM, 40 mM) **(B)** The effect of NaCl and IL-4/13 on Stat 3 and Stat3 phosphorylation was determined by Western blotting **(C)** The protein levels of STAT3, phosphorylated STAT3, FLG, and LOR were detected in HaCaT cells incubated with 40 mM NaCl and/or JAK2 inhibitor delgocitinib for 24 h **(D)** Representative immunoblotting performed to assess protein expression of LOR and FLG in keratinocytes treated with JAK2 inhibitor, NaCl, and/or IL4/13 recombinant protein **(E)** Western blotting analysis of SGK-1 and phosphorylated SGK-1 in HaCaT cells treated with the indicated concentrations of NaCl **(F)** Western blotting analysis of SGK-1 and phosphorylated SGK-1 in HaCaT cells treated with 40 mM NaCl and/or IL4/13 recombinant protein **(G)** The protein levels of SGK-1, STAT3, phosphorylated STAT3, FLG, and LOR were detected in control and SGK-1 KD HaCaT cells treated with or without 40 mM NaCl **(H)** Western blotting analysis of SGK-1, STAT3, phosphorylated STAT3, FLG, and LOR in control and SGK-1 KD HaCaT cells treated with or without 40 mM NaCl and/or IL4/13. Data are shown as mean ± standard deviation from at least three independent experiments. Statistical analysis was performed using Student's *t*-test. Data are expressed as mean ± standard deviation. ∗∗*p* < 0.01 and ∗∗∗*p* < 0.001.

In the present study, we aimed to investigate the activation of STAT3 in keratinocytes under high-salt conditions, as it is known to play a critical role in regulating keratinocyte differentiation in AD.^18^ We first performed Western blotting assays on HaCaT cells to analyze the levels of phosphorylated STAT3 when exposed to different concentrations of NaCl. The results showed a dose-dependent increase in phosphorylated STAT3, indicating that high-salt milieu activated STAT3 in keratinocytes (Fig. 3A). Next, we investigated whether there was a potential synergy between IL-4/IL-13, two canonical Th2 cytokines and established inducers of STAT3 phosphorylation in AD, and NaCl in activating STAT3. We treated HaCaT cells with IL-4/IL-13, NaCl, or a combination of both and analyzed the levels of phosphorylated STAT3. Interestingly, both IL-4/IL-13 and NaCl treatment individually up-regulated phosphorylated STAT3, and their combination had a synergistic effect on activating STAT3 (Fig. 3B). This finding suggests that NaCl induces STAT3 phosphorylation through a different mechanism than IL-4/IL-13. Further investigation into the mechanism of IL-4/IL-13-induced STAT3 activation in AD led us to the JAK–STAT3 pathway. Previous studies have reported that IL-4/IL-13 activates STAT3 through this pathway in AD.^19^ To confirm our hypothesis, we investigated the effects of delgocitinib, a pan-JAK inhibitor approved for AD treatment in Japan,^20^ on NaCl-induced STAT3 phosphorylation and blockade of keratinocyte differentiation. Western blotting analysis showed that delgocitinib partially alleviated NaCl-induced STAT3 phosphorylation and decreased the level of marker protein LOR and FLG (Fig. 3C). In addition, we performed further experiments using IL-4/IL-13 and NaCl in combination with delgocitinib. Interestingly, delgocitinib almost completely rescued STAT3 phosphorylation and keratinocyte differentiation when HaCaT cells were exposed to IL-4/IL-13 alone. However, when NaCl was added to the medium, the effects of delgocitinib were significantly reduced (Fig. 3D). This suggests that NaCl activates STAT3 in a manner different from the canonical IL-4/IL-13–JAK–STAT3 pathway.

SGK-1 is a well-characterized kinase known to be sensitive to salt in mammalian cells.^21^ To gain a deeper understanding of how NaCl inhibits keratinocyte differentiation, we conducted a study to determine whether this specific kinase mediated the effects of NaCl on HaCaT cells. Our results from Western blotting assays showed that both the expression and phosphorylation of SGK-1 increased in a dose-dependent manner with NaCl treatment (Fig. 3E). This suggests that the presence of a high-NaCl microenvironment up-regulates and activates SGK-1. Interestingly, our experiments also showed that exposure to IL-4/IL-13 did not affect the expression and activation of SGK-1 (Fig. 3F), suggesting that SGK-1 functioned as a specific target for NaCl in keratinocytes in AD. Furthermore, when we knocked down SGK-1 in HaCaT cells, it substantially reversed the NaCl-induced phosphorylation of STAT3 as well as the decrease in LOR and FLG (Fig. 3G and H). Taken together, these results demonstrate that NaCl activates the SGK-1–STAT3 signaling pathway to suppress keratinocyte differentiation in the AD microenvironment.

### SGK-1 promotes STAT3 activation via mTOR

**Figure 4:**
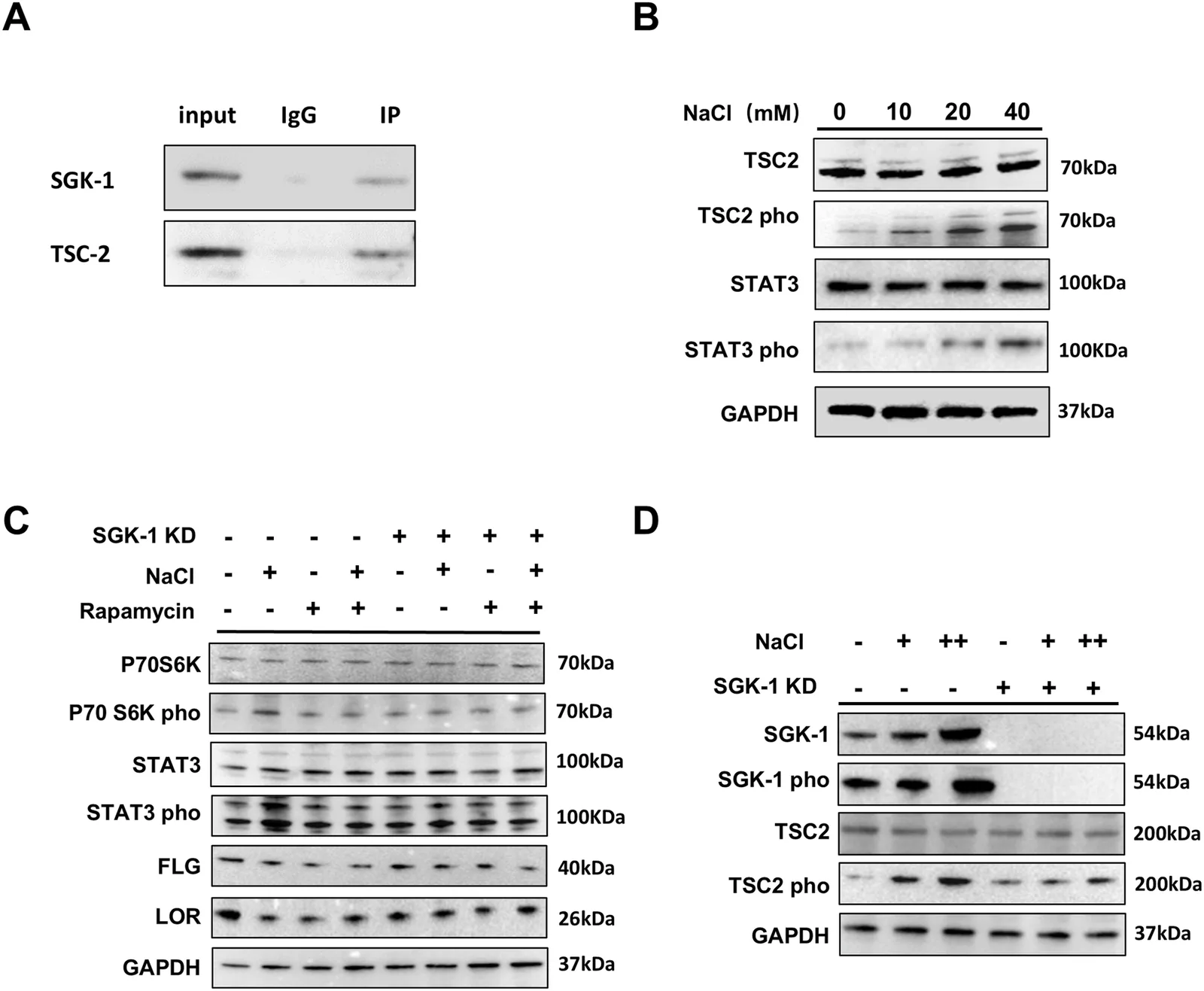
SGK-1 promotes STAT3 activation via mTOR **(A)** Immunoprecipitation analysis of TSC-2 and SGK-1 in HaCaT cells. Precipitation samples and lysates were analyzed by immunoblotting. Precipitation samples and lysates were analyzed by immunoblotting **(B)** Western blotting analysis of P70 and phosphorylated P70 protein expression levels in HaCaT cells treated with different concentrations of NaCl (0 mM, 10 mM, 20 mM, 40 mM) **(C)** The protein levels of phosphorylated STAT3, STAT3, phosphorylated STAT3, FLG, and LOR were detected in control and SGK-1 KD HaCaT cells treated with or without 40 mM NaCl and/or rapamycin **(D)** Western blotting analysis of SGK-1 and phosphorylated SGK-1 protein expression levels in control and SGK-1 KD HaCaT cells treated with or without indicated concentrations of NaCl (–0 mM, +20 mM, ++40 mM). Data are shown as mean ± standard deviation from at least three independent experiments. Statistical analysis was performed using Student's *t*-test. Data are expressed as mean ± standard deviation. ∗∗*p* < 0.01 and ∗∗∗*p* < 0.001.

To gain a better understanding of how SGK-1 modulates STAT3 activation, we performed a co-immunoprecipitation assay to investigate the direct interaction between SGK-1 and STAT3 (Fig. S3A). Surprisingly, our results demonstrated that SGK-1 did not directly bind to STAT3 in HaCaT cells, consistent with previous observations in melanoma cells.^22^ This intriguing discovery suggests the presence of another protein targeted by SGK-1 that might influence STAT3 phosphorylation. Moreover, our investigation unveiled that SGK-1 was capable of activating mTOR through the phosphorylation of TSC complex subunit 2 (TSC-2), which plays a crucial role in regulating the mTOR pathway.^23^ SGK-1 inhibits the activation of TSC2, thereby activating mTORC1 and promoting the phosphorylation of STAT3 (Fig. 4A and B). We also found that treatment with the mTOR inhibitor rapamycin successfully rescued the NaCl-induced activation of STAT3 and alleviated the blockade of keratinocyte differentiation (Fig. 4C). Furthermore, the results of our SGK-1 knockdown experiment further confirmed that the activation of STAT3 induced by NaCl occurs via the SGK-1–mTOR–STAT3 pathway (Fig. 4D).

### NaCl promotes the production of inflammatory cytokines in keratinocytes through the SGK-1–mTOR–NFκB pathway

**Figure 5:**
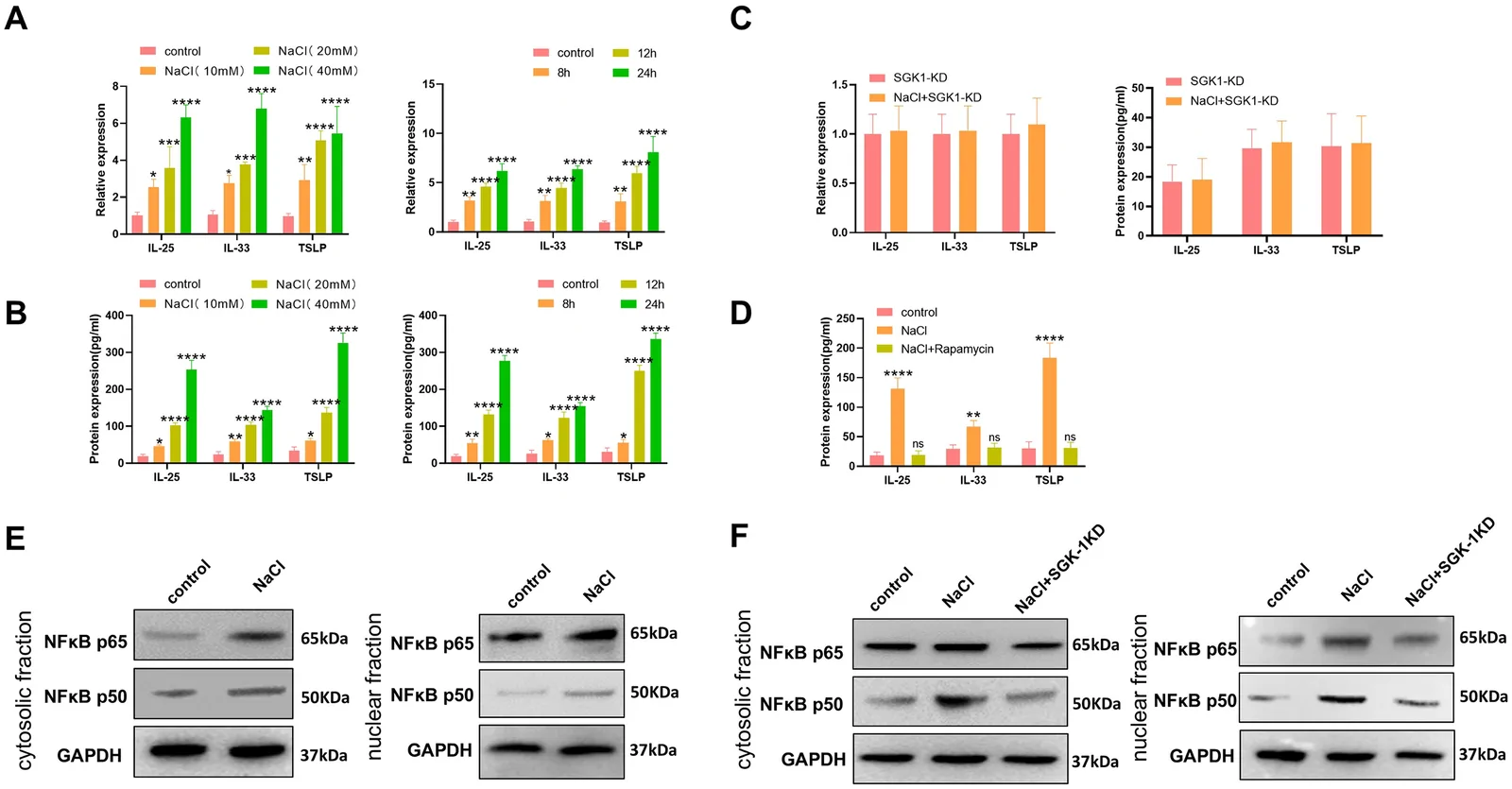
NaCl promotes the production of inflammatory cytokines in keratinocytes through the SGK-1–mTOR–NFκB pathway **(A)** Representative data of quantitative PCR analysis of IL-25, IL-33, and TSLP mRNA normalized to GAPDH in HaCaT cells treated with indicated concentrations of NaCl (0, 10, 20, 40 mM) for 24 h and 20 mM NaCl at indicated time points (0, 12 h, 24 h, 48 h). Each bar represents the mean ± standard error (*n* = 3 in each group) **(B)** HaCaT cells were treated with indicated concentrations of NaCl (0, 10, 20, 40 mM) for 24 h and 20 mM NaCl at indicated time points (0, 12 h, 24 h, 48 h), and IL-25, IL-33, and TSLP levels in the culture supernatants were measured using ELISA. Each bar represents the mean ± standard deviation of three independent experiments **(C)** Representative data of quantitative PCR analysis and ELISA analysis of IL-25, IL-33, and TSLP in control and SGK-1 KD HaCaT cells treated with 40 mM NaCl for 24 h **(D)** HaCaT cells were treated with NaCl or NaCl combined with rapamycin **(E)** HaCaT cells were treated for 24 h with or without 40 mM NaCl before Western blotting analysis of NFκB signaling molecules. Bar graphs depict the quantification of p50 and p65 in the cytosolic or nuclear fraction relative to the total amount of actin and H2B, respectively, by Western blotting densitometry **(F)** Control and SGK-1 KD HaCaT cells were treated for 24 h with or without 40 mM NaCl before Western blotting analysis of NFκB signaling molecules. Bar graphs depict the quantification of p50 and p65 in the cytosolic or nuclear fraction relative to the total amount of actin and H2B, respectively, by Western blotting densitometry. Data are shown as the mean ± standard deviation from at least three independent experiments. Statistical analysis was performed using Student's *t*-test. Data are expressed as mean ± standard deviation. ∗∗*p* < 0.01 and ∗∗∗*p* < 0.001.

Recently, keratinocytes have been found to play a critical role in the production of inflammatory cytokines that contribute to the progression of AD.^24^ Following this finding, we decided to investigate whether the high concentration of NaCl in the AD microenvironment could also affect the production of these inflammatory cytokines in keratinocytes. Our results, as shown in Figure 5A and B, demonstrated that NaCl treatment resulted in a concentration- and time-dependent increase in the mRNA and protein levels of IL-25, IL-33, and TSLP. Moreover, when SGK-1 was knocked down in HaCaT cells, the effects of NaCl on the production of these inflammatory cytokines were almost completely abolished (Fig. 5C). Furthermore, administration of rapamycin, a specific inhibitor of the mTOR pathway, was able to rescue NaCl-induced cytokine production (Fig. 5D). This suggests that the NaCl–SGK-1–mTOR pathway plays an important role in the secretion of inflammatory cytokines by keratinocytes. Nuclear factor-kappa B (NFκB) is widely recognized as a downstream target of the mTOR pathway.^25^ Therefore, we sought to determine whether NFκB was involved in this process. Our results from Western blotting assays showed that NFκB was indeed activated upon exposure to NaCl (Fig. 5E), and this activation was reduced when SGK-1 was knocked down (Fig. 5F). These results provide further evidence that NaCl promotes the secretion of inflammatory cytokines by keratinocytes through the SGK-1–mTOR–NFκB pathway.

In conclusion, our study has shown that the presence of a high concentration of NaCl in the AD microenvironment can stimulate keratinocytes to produce inflammatory cytokines. This effect is mediated by activation of the SGK-1–mTOR–NFκB pathway. These findings shed light on the pathogenesis of AD and may provide potential targets for the development of therapeutic interventions.

### Inhibiting SGK-1 alleviates NaCl-induced differentiation blockade and inflammatory cytokine production in keratinocytes

**Figure 6:**
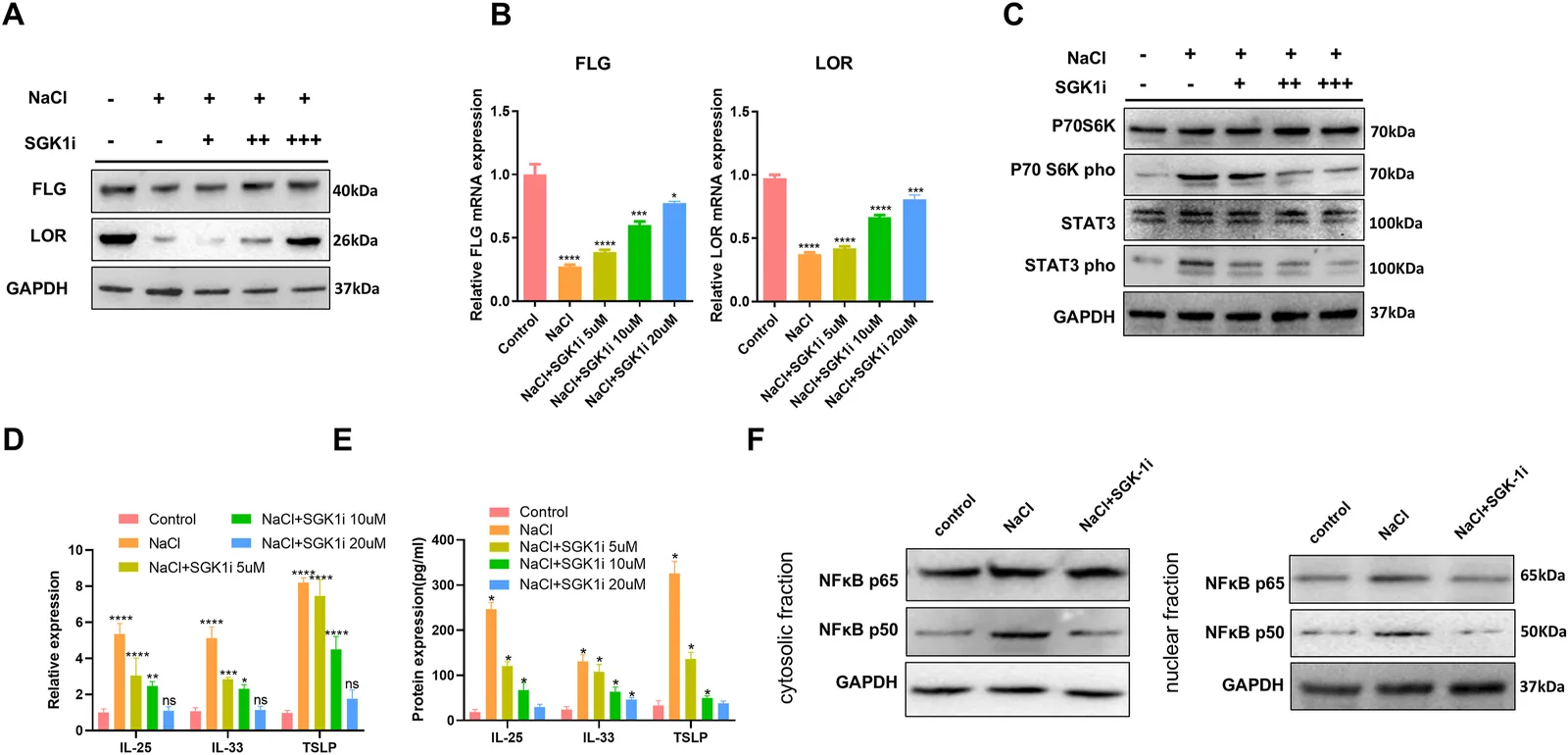
NaCl promotes the production of inflammatory cytokines in keratinocytes through the SGK-1–mTOR–NFκB pathway **(A)** Western blotting analysis of FLG and LOR in HaCaT cells treated with or without 40 mM NaCl and/or SGK-1 inhibitor GSK 650394 at indicated concentrations (–0 μM, +5 μM, ++10 μM, +++20 μM) **(B)** Quantitative PCR analysis of FLG and LOR mRNA normalized to GAPDH in HaCaT cells treated with 40 mM NaCl and/or SGK-1 inhibitor GSK 650394 at indicated concentrations (–0 μM, +5 μM, ++10 μM, +++20 μM) for 24 h **(C)** The protein levels of P70, phosphorylated P70, STAT3, and phosphorylated STAT3 in HaCaT cells treated with 40 mM NaCl and/or SGK-1 inhibitor GSK 650394 at indicated concentrations (–0 μM, +5 μM, ++10 μM, +++20 μM) for 24 h **(D)** Quantitative PCR analysis of IL-25, IL-33, and TSLP mRNA normalized to GAPDH in HaCaT cells treated with 40 mM NaCl and/or SGK-1 inhibitor GSK 650394 at indicated concentrations (0 μM, 5 μM, 10 μM, 20 μM) for 24 h **(E)** HaCaT cells were treated with 40 mM NaCl and/or SGK-1 inhibitor GSK 650394 at indicated concentrations (0 μM, 5 μM, 10 μM, 20 μM) for 24 h, and IL-25, IL-33, and TSLP levels in the culture supernatants were measured using ELISA. Each bar represents the mean ± standard deviation of three independent experiments **(F)** HaCaT cells were treated for 24 h with or without 40 mM NaCl and/or 10 μM GSK 650394 for 24 h before Western blotting analysis of NFκB signaling molecules. Bar graphs depict the quantification of p50 and p65 in the cytosolic or nuclear fraction relative to the total amount of actin and H2B, respectively, by Western blot densitometry. Data are shown as the mean ± standard deviation from at least three independent experiments. Statistical analysis was performed using Student's *t*-test. Data are expressed as mean ± standard deviation. ∗∗*p* < 0.01 and ∗∗∗*p* < 0.001.

Our research has shown that a high level of NaCl in the AD microenvironment can activate the SGK-1–mTOR pathway. This activation inhibits keratinocyte differentiation and leads to the production of inflammatory cytokines. In light of these findings, we decided to investigate whether a small molecule SGK-1 inhibitor called GSK 650394 could be repurposed as a viable treatment option for AD. To begin the study, we conducted experiments to assess the effect of GSK 650394 on rescuing NaCl-induced blockade of keratinocyte differentiation. Results from Western blotting and quantitative PCR assays indicated that GSK 650394, the SGK-1 inhibitor, was able to increase the expression of LOR and FLG at both the mRNA and protein levels in the high-salt environment (Fig. 6A and B). This suggests that the inhibitor has the potential to restore normal differentiation in keratinocytes affected by high NaCl levels. Subsequent Western blotting assays showed that the SGK-1 inhibitor was also effective in suppressing the activation of mTOR–STAT3 induced by NaCl (Fig. 6C). This further supports the potential therapeutic use of the inhibitor in the treatment of AD. Furthermore, we observed that the SGK-1 inhibitor could reduce the production of inflammatory cytokines at the mRNA and protein levels in keratinocytes exposed to NaCl (Fig. 6E and F). In addition, the inhibitor was found to inhibit the activation of NFκB, another key player in the inflammatory process (Fig. 6G). These findings suggest that the SGK-1 inhibitor can alleviate the inflammatory symptoms associated with AD.

Taken together, these results demonstrate that the SGK-1 inhibitor GSK 650394 is a promising potential drug for the treatment of AD. By effectively restoring differentiation blockade, suppressing mTOR–STAT3 activation, and reducing inflammatory cytokine production, this small molecule inhibitor may provide a rational therapeutic approach for patients suffering from AD.

### Inhibiting SGK-1 relieves AD-like symptoms *in vivo* and exerts synergized effects with delgocitinib

**Figure 7:**
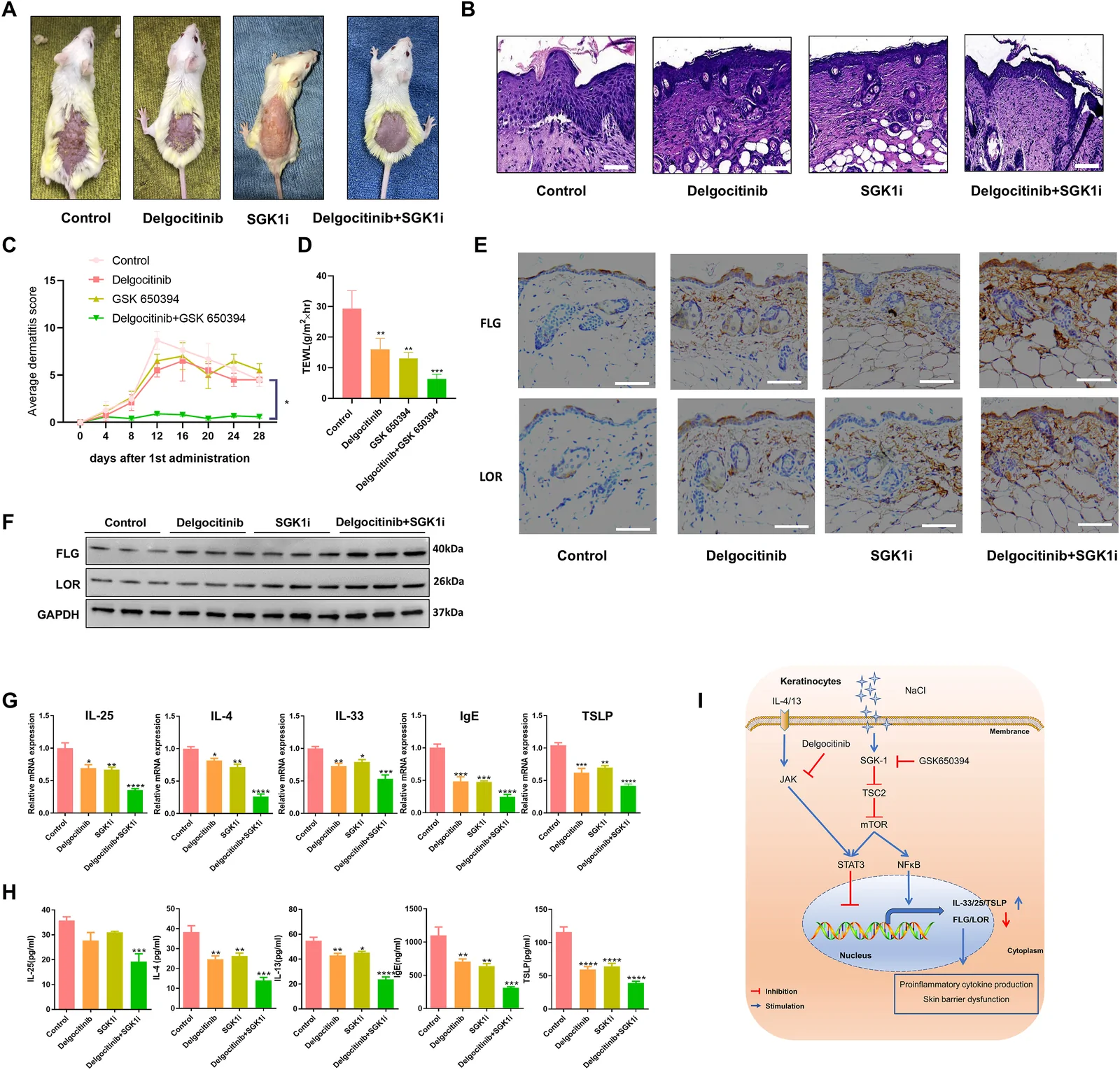
SGK-1 inhibitor relieves atopic dermatitis symptoms *in vivo* and exerts synergistic effects with JAK inhibitor **(A)** Effect of delgocitinib and/or GSK 650394 on DNCB/OVA-induced atopic dermatitis in dorsal skin **(B)** Hematoxylin-eosin staining of dorsal skin tissue sections **(C)** Effects of delgocitinib and/or GSK 650394 on dermatitis scores in DNCB-induced atopic dermatitis **(D)** Effects of delgocitinib and/or GSK 650394 on trans-epidermal water loss (TEWL) in DNCB/OVA-induced atopic dermatitis **(E)** Representative immunohistochemistry images of skin lesions for LOR and FLG in delgocitinib and/or GSK 650394-treated mice. Scale bar: 50 μm **(F)** Western blotting analysis of FLG and LOR in dorsal skin tissue of DNCB/OVA-induced atopic dermatitis mice treated with delgocitinib and/or GSK 650394 **(G)** Quantitative reverse transcription PCR assay of the related target mRNA levels in skin lesions from mice receiving different treatments. ∗*p* < 0.05, ∗∗*p* < 0.01, and ∗∗∗*p* < 0.001 **(H)** Related target molecules secretion (ELISA) of mouse model blood from different groups (*n* = 5) **(I)** Proposed working model of how NaCl modulates keratinocytes' function in atopic dermatitis. Data are shown as the mean ± standard deviation from at least three independent experiments. Statistical analysis was performed using Student's *t*-test. Data are expressed as mean ± standard deviation. ∗∗*p* < 0.01 and ∗∗∗*p* < 0.001.

To further investigate the effects of the SGK-1 inhibitor *in vivo*, we conducted experiments using mouse models of AD. The mice were divided into four groups: one group received PBS (control), another group received the SGK-1 inhibitor, a third group received the pan-JAK inhibitor delgocitinib, and the fourth group received a combination of the SGK-1 inhibitor and delgocitinib. After four weeks of treatment, we evaluated the effects of the different treatments on AD by photographing the mice and then sacrificing them for further evaluation. Upon visual inspection (Fig. 7A and B), it was evident that the skin lesions in the control group treated with PBS were more severe compared with the groups treated with delgocitinib and SGK-1 inhibitor alone. Notably, the combination therapy of SGK-1 inhibitor and delgocitinib showed the most significant improvement in AD-like symptoms. This observation was consistent with the results obtained using a scoring system called dermatitis scores and TEWL (Fig. 7C and D). In addition, we analyzed the protein expression levels by Western blotting. The results showed that the combination therapy of delgocitinib and SGK-1 inhibitor led to a significant up-regulation of FLG and LOR proteins (Fig. 7E). These proteins are known to be involved in skin barrier function, and their up-regulation suggests an improvement in skin integrity and barrier function. In addition, we examined the levels of AD-related inflammatory cytokines at both mRNA and protein levels. Our data showed that the combination therapy of SGK-1 inhibitor and delgocitinib significantly decreased the levels of IL-4, IL-25, IL-33, IgE, and TSLP (Fig. 7F and G). These cytokines play a critical role in the pathogenesis of AD, and the reduction in their levels suggests that the combination therapy has an anti-inflammatory effect.

Taken together, these results provide strong evidence that the SGK-1 inhibitor effectively suppresses the development of AD in mice and shows a synergistic effect when combined with the JAK inhibitor delgocitinib. This suggests that dual inhibition of the SGK-1 and JAK pathways may be a promising therapeutic strategy for the future treatment of AD in humans.

## Discussion

AD is a chronic inflammatory skin disease characterized by epidermal barrier dysfunction, which results from excessive inflammatory responses and leads to continuous exposure to environmental allergens.^26^^,^^27^ This creates a vicious cycle in AD pathogenesis that contributes to aggravating skin barrier deficiency.^28^ Therefore, rehabilitating skin barrier dysfunction induced by AD is increasingly recognized as a promising strategy for AD treatment.^29^ Former studies attributed AD-induced keratinocyte differentiation block mainly to a Th2 cytokine milieu in AD skin lesions.^30^ However, the AD microenvironment is a complicated ecosystem, and a variety of differences have been found to be involved in disease progression.^30^ Recently, high NaCl has been demonstrated to be a specific characteristic of skin lesions in AD.^30^ NaCl is a well-recognized immune modulating ion that can drive autoimmune disease by inducing pathogenic Th17 cells and dendritic cells via the activation of the p38/MAPK pathway and exert profound influence on the development and function of transforming growth factor-beta (TGF-β)-induced CD4^+^ Foxp3^+^ regulatory T cells.^31^, ^32^, ^33^ A recent study showed that high salt in the AD microenvironment exacerbated Th2 cell differentiation and Th2 cytokine production via an NFAT5-dependent mechanism.^16^^,^^34^ Another study reported that knockdown of the sodium channel Na_x_ could alleviate skin inflammation and improve epidermal barrier function, suggesting that NaCl may influence keratinocyte differentiation.^35^ Restoring skin barrier function is essential for the treatment of AD and can be achieved through various approaches, such as the use of topical agents (*e.g.*, emollients or lipid-based products) to repair the skin barrier and reduce water loss. There is also growing interest in the use of probiotics and prebiotics to modulate the skin microbiome and restore skin barrier function. Probiotics and prebiotics have been shown to improve skin hydration, reduce TEWL, and increase the expression of FLG and LOR.^36^ Another approach is to use immunomodulatory agents that target specific immune pathways that contribute to the development of AD. Several biological therapies targeting IgE, IL-4, or IL-13 have been approved for the treatment of moderate to severe AD. In addition, studies of the effects of high NaCl on skin barrier function and immune response are helping to advance our understanding of AD. Identifying specific immune pathways and environmental factors that contribute to the pathogenesis and exacerbation of AD will be critical to the development of effective therapies.

In this study, we present evidence that NaCl could not only affect keratinocytes but also disrupt skin barrier function and lead to the production of several inflammatory cytokines. Through further investigation, we have identified the salt-sensitive kinase SGK-1 as a key player in NaCl-induced progression of AD in keratinocytes (see Fig. 7H for a visual representation). NaCl modulates the expression of mTOR family proteins through SGK1 and activates downstream signaling pathways (Fig. S4). Our findings strongly suggest that either inhibiting NaCl uptake by cells or interfering with cellular sensing of NaCl could effectively reduce the detrimental effects of a high-salt microenvironment on skin barrier integrity and AD progression. This critical finding opens up new possibilities for therapeutic interventions that can target NaCl-related mechanisms underlying AD. Interestingly, while NaCl present in the AD microenvironment exacerbates disease progression, our experimental results with NaCl administered via drinking water showed an opposite effect. Indeed, we observed that NaCl intake via drinking water not only failed to promote AD progression but actually provided some relief from AD-like symptoms, as shown in Figure S5. This intriguing finding could be attributed to the regulation of the gut microbiome induced by NaCl intake, as previous research has shown that a high-salt diet can stimulate potent anti-tumor immunity by modulating the microbiota.^37^ Thus, our findings suggest that AD patients should not deliberately restrict their NaCl intake from dietary sources. Instead, an essential intake of NaCl may potentially offer benefits in alleviating their symptoms. This unexpected finding highlights the complex interplay between NaCl, the gut microbiome, and the pathophysiology of AD and calls for further investigation to fully understand these intricate interactions.

Keratinocytes, the major cell type in the epidermis, undergo a highly regulated process of differentiation to form a structured hierarchical epidermal layer.^38^ However, the specific mechanism by which AD promotes a block in keratinocyte differentiation is only partially understood. Previous studies have shown that the presence of Th2 cytokines in the AD microenvironment leads to the activation of JAK, which in turn phosphorylates STAT3. In addition, IL-33, a cytokine released during AD inflammation, has been shown to promote the phosphorylation of STAT3 by forming a complex with it. Once phosphorylated, STAT3 translocates to the nucleus of keratinocytes and inhibits their differentiation process.^18^^,^^19^^,^^39^ In addition, recent research has identified the transcription factor p63 as a critical factor that responds to another cytokine, IL-4/IL-13, present in the AD microenvironment. This interaction between IL-4/IL-13 and p63 leads to an inhibition of the differentiation of keratinocytes, further contributing to the skin barrier dysfunction observed in AD.^40^ In this study, we present novel evidence implicating the SGK-1–mTOR–STAT3 signaling pathway in the development of skin barrier dysfunction in AD. We show that SGK-1, a serine/threonine kinase, contributes significantly to the activation of STAT3 and the subsequent impairment of keratinocyte differentiation. Importantly, we identify an SGK-1 inhibitor as a potential therapeutic option for AD. Unlike JAK inhibitors that target only the JAK–STAT pathway, the SGK-1 inhibitor acts independently of JAK activation, suggesting that it could be used in combination with JAK inhibitors to achieve a more satisfactory clinical outcome. Our combination therapy using both the SGK-1 inhibitor and JAK inhibitors shows promising results in murine models of AD. It effectively alleviates AD-like symptoms in murine models and has great potential for future clinical use in AD patients. In conclusion, our study sheds light on the intricate mechanisms underlying the blockade of differentiation and inflammatory cytokine production of keratinocytes induced by a high-salt microenvironment in AD. The discovery of the SGK-1–mTOR–STAT3 pathway and the potential of an SGK-1 inhibitor as a therapeutic modality provide new avenues for the management and treatment of AD.

Taken together, in this study, we have discovered that within the context of AD, a high-NaCl microenvironment restrains the differentiation of keratinocytes, exacerbates the generation of inflammatory cytokines, and advances the progression of the disease through the activation of the SGK-1–mTOR pathway. Furthermore, we have successfully identified an inhibitor of SGK-1 that can effectively overturn the impediment to keratinocyte differentiation and cytokine production induced by NaCl, both *in vitro* and *in vivo*. Additionally, when combined with a JAK inhibitor, this inhibitor exhibits a synergistic effect in alleviating the symptoms of AD.

## CRediT authorship contribution statement

**Yingqiang Luo:** Formal analysis, Data curation. **Zhiqiang Song:** Writing – review & editing, Methodology, Conceptualization. **Pengju Jiang:** Writing – original draft, Data curation. **Lan Ge:** Visualization, Investigation. **Min Zhang:** Writing – original draft, Data curation. **Zihao Zhou:** Writing – original draft, Data curation. **Yaguang Wu:** Writing – original draft, Methodology, Funding acquisition, Data curation, Conceptualization. **Jun Hu:** Writing – review & editing, Funding acquisition, Formal analysis, Data curation.

## Data availability

The original contributions presented in the study are included in the article/supplementary material, and further inquiries can be directed to the corresponding author.

## Funding

This work was supported by the 10.13039/501100001809National Natural Science Foundation of China (No. 82304032), the 10.13039/501100005230Natural Science Foundation of Chongqing, China (No. CSTB2023NSCQ-MSX1081), and the Science and Technology Research Program of Chongqing Education Commission of China (No. KJQN202312808, KJZD-k202312804).

## Conflict of interests

The authors declare that the research was conducted in the absence of any commercial or financial relationships that could be construed as a potential conflict of interests.

## References

[1] Bieber T. (2022). Atopic dermatitis: an expanding therapeutic pipeline for a complex disease. Nat Rev Drug Discov.

[2] Langan S.M., Irvine A.D., Weidinger S. (2020). Atopic dermatitis. Lancet.

[3] Laughter M.R., Maymone M.B.C., Mashayekhi S. (2021). The global burden of atopic dermatitis: lessons from the Global Burden of Disease Study 1990-2017. Br J Dermatol.

[4] Ständer S. (2021). Atopic dermatitis. N Engl J Med.

[5] Narla S., Silverberg J.I. (2020). The role of environmental exposures in atopic dermatitis. Curr Allergy Asthma Rep.

[6] Woo Y.R., Park S.Y., Choi K., Hong E.S., Kim S., Kim H.S. (2020). Air pollution and atopic dermatitis (AD): the impact of particulate matter (PM(10)) on an AD mouse-model. Int J Mol Sci.

[7] Butala S., Castelo-Soccio L., Seshadri R. (2023). Biologic versus small molecule therapy for treating moderate to severe atopic dermatitis: clinical considerations. J Allergy Clin Immunol Pract.

[8] Goleva E., Berdyshev E., Leung D.Y. (2019). Epithelial barrier repair and prevention of allergy. J Clin Invest.

[9] Lefèvre-Utile A., Braun C., Haftek M., Aubin F. (2021). Five functional aspects of the epidermal barrier. Int J Mol Sci.

[10] Gallegos-Alcalá P., Jiménez M., Cervantes-García D., Salinas E. (2021). The keratinocyte as a crucial cell in the predisposition, onset, progression, therapy and study of the atopic dermatitis. Int J Mol Sci.

[11] Bieber T. (2012). Atopic dermatitis 2.0: from the clinical phenotype to the molecular taxonomy and stratified medicine. Allergy.

[12] Moosbrugger-Martinz V., Leprince C., Méchin M.C. (2022). Revisiting the roles of filaggrin in atopic dermatitis. Int J Mol Sci.

[13] Chieosilapatham P., Kiatsurayanon C., Umehara Y. (2021). Keratinocytes: innate immune cells in atopic dermatitis. Clin Exp Immunol.

[14] Ito T., Wang Y.H., Duramad O. (2005). TSLP-activated dendritic cells induce an inflammatory T helper type 2 cell response through OX40 ligand. J Exp Med.

[15] Han N.R., Oh H.A., Nam S.Y. (2014). TSLP induces mast cell development and aggravates allergic reactions through the activation of MDM2 and STAT6. J Invest Dermatol.

[16] Matthias J., Maul J., Noster R. (2019). Sodium chloride is an ionic checkpoint for human T(H)_2_ cells and shapes the atopic skin microenvironment. Sci Transl Med.

[17] Jiang P., Wu Y., Liu L., Zhang L., Song Z. (2022). Combined application of dinitrofluorobenzene and ovalbumin induced AD-like dermatitis with an increase in helper T-cell cytokines and a prolonged Th2 response. BMC Immunol.

[18] Dai X., Muto J., Shiraishi K. (2022). TSLP impairs epidermal barrier integrity by stimulating the formation of nuclear IL-33/phosphorylated STAT3 complex in human keratinocytes. J Invest Dermatol.

[19] Amano W., Nakajima S., Kunugi H. (2015). The Janus kinase inhibitor JTE-052 improves skin barrier function through suppressing signal transducer and activator of transcription 3 signaling. J Allergy Clin Immunol.

[20] Saeki H., Ohya Y., Furuta J. (2022). Executive summary: japanese guidelines for atopic dermatitis (ADGL) 2021. Allergol Int.

[21] Lang F., Böhmer C., Palmada M., Seebohm G., Strutz-Seebohm N., Vallon V. (2006). (Patho)physiological significance of the serum- and glucocorticoid-inducible kinase isoforms. Physiol Rev.

[22] Araki T., Watanabe Y., Okada Y., Murakami H., Ogo N., Asai A. (2022). Identification of serum and glucocorticoid-regulated kinase 1 as a regulator of signal transducer and activator of transcription 3 signaling. Exp Cell Res.

[23] Li Y., Corradetti M.N., Inoki K., Guan K.L. (2004). TSC2: filling the GAP in the mTOR signaling pathway. Trends Biochem Sci.

[24] Das P., Mounika P., Yellurkar M.L. (2022). Keratinocytes: an enigmatic factor in atopic dermatitis. Cells.

[25] Xu T., Liu J., Li X.R. (2021). The mTOR/NF-κB pathway mediates neuroinflammation and synaptic plasticity in diabetic encephalopathy. Mol Neurobiol.

[26] Cook-Mills J.M., Emmerson L.N. (2022). Epithelial barrier regulation, antigen sampling, and food allergy. J Allergy Clin Immunol.

[27] Leung D.Y.M., Berdyshev E., Goleva E. (2020). Cutaneous barrier dysfunction in allergic diseases. J Allergy Clin Immunol.

[28] Egawa G., Kabashima K. (2016). Multifactorial skin barrier deficiency and atopic dermatitis: essential topics to prevent the atopic March. J Allergy Clin Immunol.

[29] Yang X., Kambe N., Takimoto-Ito R., Kabashima K. (2021). Advances in the pathophysiology of atopic dermatitis revealed by novel therapeutics and clinical trials. Pharmacol Ther.

[30] Dainichi T., Kitoh A., Otsuka A. (2018). The epithelial immune microenvironment (EIME) in atopic dermatitis and psoriasis. Nat Immunol.

[31] Kleinewietfeld M., Manzel A., Titze J. (2013). Sodium chloride drives autoimmune disease by the induction of pathogenic TH17 cells. Nature.

[32] Luo Y., Xue Y., Wang J. (2019). Negligible effect of sodium chloride on the development and function of TGF-β-induced CD4^+^ Foxp3^+^ regulatory T cells. Cell Rep.

[33] Xiao Z.X., Hu X., Zhang X. (2020). High salt diet accelerates the progression of murine lupus through dendritic cells via the p38 MAPK and STAT1 signaling pathways. Signal Transduct Target Ther.

[34] Zielinski C.E. (2021). Regulation of T cell responses by ionic salt signals. Cells.

[35] Zhao J., Jia S., Xie P. (2020). Knockdown of sodium channel Na_x_ reduces dermatitis symptoms in rabbit skin. Lab Invest.

[36] Kim S., Han S.Y., Lee J. (2022). *Bifidobacterium longum* and galactooligosaccharide improve skin barrier dysfunction and atopic dermatitis-like skin. Allergy Asthma Immunol Res.

[37] Rizvi Z.A., Dalal R., Sadhu S. (2021). High-salt diet mediates interplay between NK cells and gut microbiota to induce potent tumor immunity. Sci Adv.

[38] Rice G., Rompolas P. (2020). Advances in resolving the heterogeneity and dynamics of keratinocyte differentiation. Curr Opin Cell Biol.

[39] Dai X., Shiraishi K., Muto J. (2022). Nuclear IL-33 plays an important role in IL-31‒mediated downregulation of FLG, keratin 1, and keratin 10 by regulating signal transducer and activator of transcription 3 activation in human keratinocytes. J Invest Dermatol.

[40] Brauweiler A.M., Leung D.Y.M., Goleva E. (2021). The transcription factor p63 is a direct effector of IL-4- and IL-13-mediated repression of keratinocyte differentiation. J Invest Dermatol.

