# Supplementary material for: High-salt microenvironment worsens the progression of atopic dermatitis via activating the SGK–1–mTOR pathway in keratinocytes
Source: Genes Dis. 2026 Mar 27;13(6):102168. doi: 10.1016/j.gendis.2026.102168 (PMC13499190; doi:10.1016/j.gendis.2026.102168)
Supplement: Supplementary file 1 [file mmc1.docx]

**Supplementary figures**

**Figure S1** Analysis of cell viability and apoptosis. HaCaT cells were treated with or without Mannitol, NaCl, Na-gluconate, and Choline-Cl for 24 hours, after which cell viability and apoptosis levels were assessed. (A) Cell viability was determined using the cell counting kit-8. (B) Apoptosis levels were evaluated by flow cytometry following Annexin V-FITC/PI staining of the HaCaT cells.

**Figure S2** Impact of NaCl on skin barrier function. Following treatment with isotonic solutions, the expression levels of osmotic pressure and skin barrier proteins were assessed by western blot.

**Figure S3** Immunoprecipitation analysis of STAT3 and SGK-1 in HaCaT cells treated with the NaCl for 72 h. Precipitation samples and lysates were analyzed by immunoblotting. Precipitation samples and lysates were analyzed by immunoblotting.

**Figure S4** Role of NaCl in regulating the SGK-1-mTOR-STAT3/NFκB axis After treatment of HaCaT cells, protein expression levels were analyzed by western blot to strengthen the causal chain.

**Figure S5** NaCl in drinking has limited influence on AD symptoms in vivo. (A) Representative photos of dorsal skin in DNCB/OVA-induced atopic dermatitis in PBS and NaCl treated mice. (B) Representative mice model skin tissues are shown by HE staining. (C) Time course of total clinical severity scores of PBS and NaCl treated mice (n=5).

**Table S1** Reagent

| **Antibody** | **Catalog** | **Company** |
| --- | --- | --- |
| FLG | sc-57133 | CSBT |
| LOR | PA5-30583 | Thermofisher |
| GAPDH | 380646 | Shener |
| Phospho-SGK1 | AF3001 | Affinity |
| SGK1 | 52499R | Bioss |
| phospho-STAT3 | 22386R | Bioss |
| STAT3 | 55208R | Bioss |
| NFκB p65 | 33117M | Bioss |
| NFκB p50 | 1194R | Bioss |
| TSC2 | MA5-15004 | Thermofisher |
| Phospho-TSC2 | 710395 | Thermofisher |
| p70 | 701261 | Thermofisher |
| Phospho-p70 | 710095 | Thermofisher |
